# Current Evidence of Ergogenic and Post-Exercise Recovery Effects of Dietary Supplementation with *Cordyceps militaris* in Humans—A Narrative Review

**DOI:** 10.3390/nu18050781

**Published:** 2026-02-27

**Authors:** Maciej Jędrejko, Karol Jędrejko, Dominika Granda, Katarzyna Kała, Andrzej Pokrywka, Bożena Muszyńska

**Affiliations:** 1 Department of Medicinal Plant and Mushroom Biotechnology, Medical College, Jagiellonian University, 9 Medyczna Street, 30-688 Kraków, Poland; maciej.jedrejko@proton.me (M.J.); karol.jedrejko@gmail.com (K.J.); k.kala@uj.edu.pl (K.K.); 2 Department of Nutrition Physiology, Institute of Sport-National Research Institute, 01-982 Warsaw, Poland; dominika.granda@insp.pl; 3 Department of Biochemistry and Pharmacogenomics, Faculty of Pharmacy, Medical University of Warsaw, 02-097 Warsaw, Poland; andrzej.pokrywka@wum.edu.pl

**Keywords:** *Cordyceps militaris*, cordycepin, ergothioneine, ergogenic, exercise capacity, performance, post-exercise recovery

## Abstract

*Cordyceps militaris* is an entomopathogenic fungus traditionally used in Asian ethnomedicine and increasingly investigated for its potential health-promoting properties, including immunomodulatory and anti-inflammatory activities. In recent years, it has gained attention as a dietary supplement with possible applications in sports nutrition. This narrative review summarizes and critically evaluates the current human evidence regarding the ergogenic and post-exercise recovery effects of *C. militaris* supplementation in healthy individuals. A structured database search was conducted using predefined eligibility criteria, and the methodological quality of included studies was appraised through domain-based risk-of-bias assessment. Five intervention studies published between 2017 and 2024, comprising 321 participants aged 16–35 years, were identified. Supplementation protocols ranged from 1 to 16 weeks, with daily doses of 1–12 g administered either as isolated fungal material or as a part of multi-ingredient formulations. Assessed outcomes included indices of aerobic performance and exercise capacity, such as maximal or peak oxygen uptake (VO_2_max/VO_2_peak), time to exhaustion, power output, running performance, and maintenance of peripheral oxygen saturation during high-intensity exercise. Several studies also evaluated biochemical markers related to muscle damage and inflammatory responses, including creatine kinase, blood urea nitrogen, and white blood cell counts. Although some studies reported improvements in selected performance and recovery parameters, the findings were inconsistent. The certainty of the evidence is limited by small sample sizes, heterogeneity of participants and exercise protocols, insufficient reporting of randomization, lack of trial registration in most studies, absence of standardized preparations with quantified bioactive constituents, and the use of multi-ingredient supplements. Well-designed randomized controlled trials using chemically characterized preparations and homogeneous athletic populations are required to clarify the efficacy and practical relevance of *C. militaris* in sports nutrition.

## 1. Introduction

The concept of an ergogenic aid, originally defined in the late sixties of the 20th century as a means of enhancing physical and mental exercise capacity while reducing fatigue, has evolved over time. Contemporary recommendations regarding performance-enhancing nutritional strategies are now based on evidence-informed guidelines developed by leading sports organizations, including the International Olympic Committee, the International Society of Sports Nutrition, and the Australian Institute of Sport [1,2,3,4,5].

The use of natural substances to enhance performance, however, dates back to antiquity. Ancient Greek athletes reportedly consumed plant products and raw mushrooms to improve strength, speed, focus, and pain tolerance. In some instances, toxic mushroom species were reportedly consumed for their psychoactive properties, which may have influenced arousal and perception through cholinergic bioactive compounds exerting muscarinic-like effects [6,7,8].

**Figure 1:**
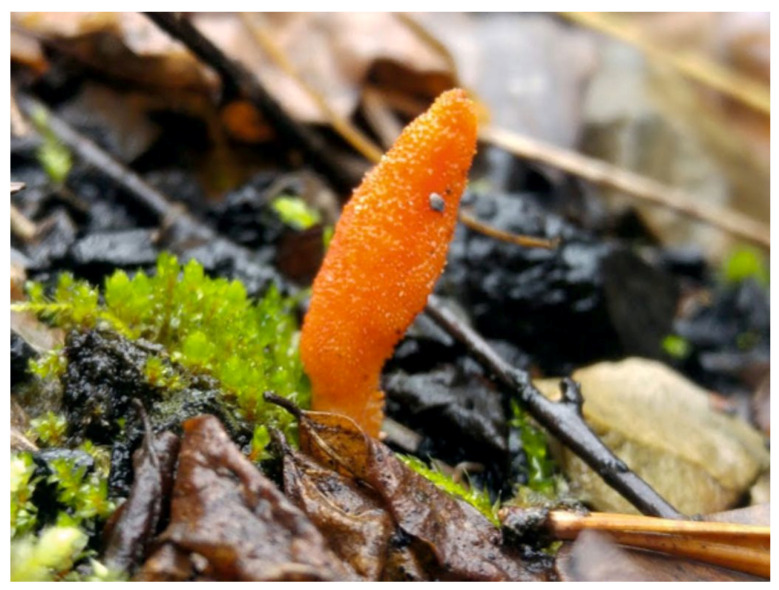
Naturally growing *Cordyceps militaris* (photography by Paweł Stasiowski).

The genus *Cordyceps* includes more than 700 different species, and some of the popular species are *Cordyceps sinensis* (currently named *Ophiocordyceps sinensis*, hereafter referred to as *O. sinensis*) and *Cordyceps militaris*. *C. militaris* (Figure 1) is found in lower altitude regions, typically within 1000–2000 m MSL [9]. Although the European Union (EU) does not classify *C. militaris* as an edible mushroom, this mushroom species is recognized as a medicinal mushroom. In EU countries, it is categorized as an unauthorized ingredient of foods and dietary supplements in accordance with the European Commission (EC) and European Food Safety Authority (EFSA) novel food catalog and is recognized as unauthorized novel food [10].

Various *Cordyceps* species have been used for centuries in Asian traditional medicine (China, India, Japan, Korea) to support adaptation to extreme high-mountain environments (low temperature, hypoxia). These fungal species are recognized for their natural anti-hypoxic activity, which helps alleviate fatigue, enhance both physical and mental conditions, and strengthen the immune system [11,12,13]. According to recent studies, *C. militaris* shows several health-beneficial effects in humans, including immunostimulatory effects [14,15] and as a supporting agent for treating chronic bronchitis [16]. These properties are attributed to the rich content of numerous chemical components, including amino acids, proteins, carotenoids, phenolic acids, nucleosides, polysaccharides, and steroids [9,17].

Previous studies conducted on animal models show that *C. militaris* intake has beneficial effects on exercise performance, exercise capacity, and post-exercise recovery [18,19,20]. Although the results of these studies are promising, they do not translate directly to the use of *C. militaris* in humans. One of the main reasons for this is the aforementioned rich composition of diverse active ingredients. On the one hand, the mechanism of action of some of them on the human body is still not fully understood. On the other hand, *C. militaris* is not particularly promoted among physically active people and athletes, and, in addition, it does not appear in any official recommendations. Currently, very few scientific studies have been conducted on *Cordyceps* supplementation in the context of exercise performance, and unfortunately, their results have been inconsistent.

This narrative review aims to summarize and appraise the current evidence from human trials investigating the impact of *C. militaris* supplementation on aerobic/anaerobic performance metrics and post-exercise recovery in healthy, physically active populations.

## 2. Research Selection Criteria

The following three electronic databases were searched: PubMed, Scopus, and Web of Science, and a complementary search was conducted on Google Scholar. The search was conducted for all records available from the inception of each database up to 10 December 2025. The following keywords were used for search: “cordyceps,” “cordycepin,” “ergogenic,” “exercise,” “performance,” “endurance,” “oxygen saturation,” “oxygen consumption,” “anti-fatigue,” “physical loads,” and “muscle strength.” These keywords were combined with Boolean operators (“AND” and “OR”).

The following inclusion criteria were considered: (1) interventional studies in healthy individuals (i.e., without chronic diseases) who were administered *C. militaris*; (2) studies in which participants had to perform exercises/were subjected to physical exertion; and (3) publications in English.

The exclusion criteria were as follows: (1) poster presentations, conference abstracts, and review articles; (2) in vitro studies, animal studies, and observational or other non-interventional studies; (3) studies involving children (i.e., under 16 years of age) or pregnant women; (4) studies involving medical treatment; and (5) studies that did not report outcomes related to endurance or exercise performance.

Three authors (D.G., K.J., and M.J.) independently assessed the titles and abstracts of the screened studies to determine their eligibility for inclusion in this review. The full texts were thoroughly evaluated based on predefined inclusion and exclusion criteria. Discrepancies were resolved through discussion with other co-authors (K.K., B.M., and A.P.).

In cases where necessary information was missing from potentially eligible studies, the required details were acquired by contacting the specific author. The following data were extracted from the studies: authors’ names; study design; and number, age, and gender of participants. Additionally, the following information regarding the methodology or results of the study was retrieved: duration of intervention and dose and characteristics of the mushroom material used. Funding details and safety assessment data (reporting adverse effects during the study) were also obtained.

Two authors (K.J. and M.J.) independently assessed the quality of the studies by using Cochrane Collaboration’s Risk of Bias tool (RoB 2) [21].

All studies were analyzed according to the following criteria: blinding of participants, personnel, and outcome assessment; random sequence generation; allocation concealment; incompleteness of data obtained; selectivity of reporting results; and other potential sources of bias. To resolve discrepancies, a thorough re-analysis was conducted to reach consensus and eliminate any doubts. Furthermore, any concerns were discussed with senior authors.

Each study was assessed based on five distinct domains: (1) bias due to randomization process; (2) bias due to deviations from intended interventions; (3) bias due to missing outcome data; (4) bias in outcome measurement; and (5) bias in the selection of reported results. The overall risk of bias was categorized as low (green), moderate (yellow), and high (red).

## 3. Ergogenic and Post-Exercise Recovery Effect of Dietary Supplementation with *Cordyceps militaris* in Humans

### 3.1. Description of the Included Studies

**Figure 2:**
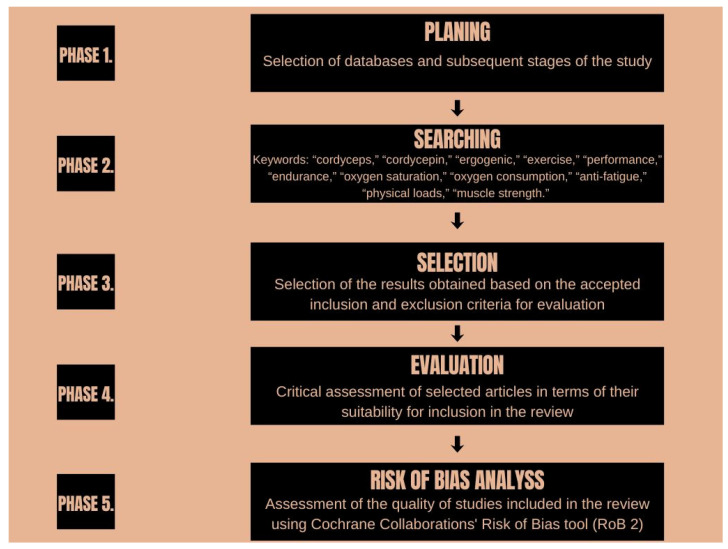
Diagram of review methodology.

Figure 2 shows the process of selecting studies.

The initial multi-database search yielded 1825 records that corresponded to the following results: 500 from PubMed, 457 from Scopus, and 868 from Web of Science. After removing 548 duplicate records, 1277 publications were checked for the title and abstract. Based on title and abstract check, 1162 publications were excluded because they were not related to *C. militaris* (982 articles) or reported medical treatment (37 articles), analytical tests (68 articles), in vitro studies (16 articles), or animal studies (57 articles). In addition, two review articles were excluded. Finally, the remaining 115 publications were subjected to full text eligibility assessment.

Based on full-text analysis, 113 papers were excluded because they reported results not related to physical activity and exercise capacity. Thus, from the database search, only two studies were considered eligible for inclusion in this review. Next, a manual search of Google Scholar was conducted. Based on the keywords and inclusion criteria, three additional studies were identified. Finally, five studies (*n* = 5) were included in this narrative review.

These five scientific studies were published between 2017 and 2024. A total of 321 individuals of both sexes, aged 16–35 years, participated in these studies. The dose of products containing *C. militaris* ranged from 1 to 12 g per day, with both pure fungal materials and multi-ingredient dietary supplements used in the studies. The dietary supplementation duration was 1 to 16 weeks.

### 3.2. Characteristics of the Studies

The specific data from each study included in this review are presented in Table 1.

In two of the five studies from Table 1, PeakO_2_ was determined following consumption of a blend of six mushrooms, with *C. militaris* as the main ingredient. Hirsch et al. [22] demonstrated that a 3-week dietary intervention of PeakO2^®^ mushroom blend (primary ingredient: *C. militaris*) at the dose of 4 g per day improves tolerance to high-intensity exercise. However, the intake of this blend for 1 week did not significantly affect aerobic and anaerobic exercise performance in young participants. In the study of Dudgeon et al. [23], the effect of PeakO2^®^ on the selected exercise parameters of healthy individuals (aged 19–34 years) was examined at a low dose (1–2 g/day) and a high dose (12 g/day) for 4 weeks and 1 week, respectively. Small doses administered over a long period of time reduced blood lactate (BL) levels, increased time to exhaustion (TTE), and increased VO_2_peak. In contrast, high doses administered for a shorter period were more beneficial for the less-trained participants.

Wang [24] examined the effect of *C. militaris* in a group of 180 young swimmers of both sexes (aged 16–18 years). The experimental group participants were individuals with over 5 years of experience who underwent a 7-week training period. The swimmers had 6 training days each week, with 4 training sessions per day (2 in the morning and 2 in the evening; total: 8 h/day). Some participants took 8 g of *C. militaris* (boiled in 300 mL of water) daily as a supplement to their dinner. After 1, 3, and 7 weeks of training and supplementation, blood samples were analyzed. Swimmers taking *C. militaris* had higher levels of hemoglobin (Hb), white blood cells (WBC), and testosterone (T) and lower levels of creatine kinase (CK) and blood urea nitrogen (BUN). These results show that *C. militaris* supplementation was beneficial during post-exercise recovery, e.g., it provided greater post-exercise resistance to muscle damage. The beneficial effect on the body’s immune response to physical exertion was confirmed by changes in IL-4 and IFN-γ levels. Swimmers taking *C. militaris* had lower IL-4 levels after 7 weeks, indicating less severe inflammatory reactions induced by training. Additionally, a more intense increase in IFN-γ values shows that regular *C. militaris* supplementation induces stronger regenerative processes. Swimmers also underwent a 30 s maximal effort cycle test to assess the effect of *C. militaris* on power and BL levels. Although the lactate concentration of the supplementation group did not differ from that of the control group, swimmers taking *C. militaris* had significantly higher maximum and average power values.

A group of 22 long-distance runners (aged 18–24 years) underwent a 16-week supplementation protocol during their pre-season training. Eleven participants took 1800 mg of *C. militaris* mycelium extract (6 capsules × 300 mg) daily, while the other participants took the same amount of placebo (potato starch capsules). The groups showed no significant differences in distance covered and running time. However, significant changes were noted in the blood test results. After 16 weeks of *C. militaris* use, the participants showed lower WBC counts and a significant reduction in CK levels, one of the key markers of muscle damage due to physical exertion. This finding suggests that, in long-distance runners, *C. militaris* extract may be effective as a pro-regenerative component that eliminates the adverse effects of intense training sessions [25].

In the study of Pasha et al. [26], 48 young male and female runners (aged 16–35 years) were recruited and trained for 1–5 years under the supervision of an Olympic coach. The participants were assigned to four groups with the following consumption regimen for 3 weeks: 150 mL of black tea (placebo), 150 mL of black tea + 10 g of whey protein, 1 g of freshly ground *C. militaris* fruiting bodies + 150 mL of black tea, or 1 g of freshly ground *C. militaris* fruiting bodies + 150 mL of black tea + 10 g of whey protein. After a 3-week supplementation protocol, the runners underwent exercise tests: a 200 m and 5 km run, and a 3 min maximum treadmill run test. The participants taking *C. militaris* showed a high degree of oxygen saturation (93–95%, respectively), while the other participants experienced a sharp decrease in the degree of oxygen saturation (60–75%). The *C. militaris* infusion group showed a significant reduction in the time required to cover distances of 200 m and 5 km compared to the control group. Thus, the inclusion of *C. militaris* in the diet has beneficial effects on physically active people, although it should be noted that the improvement level was lower when freshly ground fruiting bodies were combined with whey protein.

### 3.3. Risk of Bias Analysis

The results of the risk of bias analysis are presented in Table 2.

None of the analyzed studies had an overall low risk of bias. Four studies [23,24,25,26] were not registered in clinical trial databases and did not have a registration number, making it difficult to ensure data completeness. Thus, according to the Cochrane Collaboration’s RoB 2 criteria [21], they were assigned a high risk of bias. The remaining fifth study [22] was registered, although there was a risk of result omission at the second stage with significantly fewer participants. Therefore, we assumed that this study had a moderate risk of bias.

A noteworthy finding is that the outcome measurement showed a low risk of bias in four studies [22,23,25,26]. Although all studies involved objective physiological parameters, which were measured using standardized methods or equipment, Wang’s study [24] lacked precise information regarding the randomization and blinding processes for those conducting the measurement procedures.

The study of Pasha et al. [26] is distinct from other studies, wherein the authors ensured minimization of the risk of bias in other aspects. The study was a double-blinded investigation, with all participants randomly assigned to four groups using a recognized software. The authors collected baseline data and reported no baseline imbalances. The infusion samples were not only coded but also made visually and sensorily similar. During the study, there were no deviations from the planned protocol, as the codification and similarities of the infusion samples reduced the risk of systematic errors. All participants completed the study, and there were no dropouts; moreover, the data before and after the experiment were obtained for each group of participants. This study can be considered a model for future work to assess the ergogenic effects of *C. militaris* in humans. A detailed discussion of the quality assessment of other studies is presented in Supplementary Materials.

This narrative review further expands on issues explored in a recent publication by Dewi and Khemtong [27] on the ergogenic properties of *Cordyceps* mushrooms.

The results of this review indicate that *C. militaris* actually exerts a beneficial, ergogenic effect on physical activity. Among participants using preparations containing *C. militaris*, improvements were observed in exercise capacity parameters (VO_2max_, TTE, SpO_2_, running time, and P_max_) together with a substantial reduction in the markers of muscle damage (e.g., BUN and CK). Another noteworthy aspect is that the obtained results were derived from investigative studies based on the inclusion criteria used in the methodology, wherein conference materials such as abstracts and posters were excluded because they do not allow for a detailed analysis of the results and present limited data. Hirsch et al. [28] recruited a group of healthy young individuals (recreationally active) in their study and reported an improvement of selected exercise parameters such as VO_2max_ and TTE in high-intensity aerobic exercise following a 3-week supplementation of a mushroom blend (4 g daily) containing *C. militaris* as the primary ingredient. Moreover, a 2-week supplementation with a combination of *C. militaris*, arginine, and citrulline (2 g/day of each ingredient) markedly enhanced running economy and performance in a group of 21 male and female runners who underwent a 45 min incremental exercise test under hot conditions [29].

## 4. Strengths and Limitations of the Review

The main strength of this narrative review lies in the comprehensive and transparent approach to identifying, reviewing, and synthesizing the available literature. The review of the databases, initial selection of studies to be included in the review, and quality assessment of the included studies were conducted independently by three researchers. A risk of bias analysis was also performed to assess the reliability of the methodological procedures and confirm the credibility of the obtained results.

One of the main limitations of this review is that four of the five analyzed studies exhibited methodological concerns. This issue might be a cause of concern because, although the results indicate positive effects of *C. militaris* use among participants, the outcomes might have been influenced by limitations in the methodology and research protocol.

Moreover, beyond these methodological concerns, the publications included in this review also have other significant limitations. The first and most significant limitation is the fungal material used. None of the studies used a standardized product with labeled active ingredients. *C. militaris* contains many bioactive components with beneficial effects on physical activity. These bioactive components include cordycepin (3′-adeoxyadenosine), ergothioneine, and polysaccharides [30]. This is a critical concern because studies conducted on animal models show that all these components had a positive effect on aspects related to physical performance and post-exercise recovery [19,31].

Another limitation is that two [22,23] of the five studies used a multicomponent preparation in which *C. militaris* was one of the several components. Although the authors of these two studies specified the amount of *C. militaris* per daily dose in the supplementation protocol, no information was provided regarding the content of the other mushrooms in the mixture. The use of a multi-ingredient supplement prevents a reliable assessment of the direct impact of *C. militaris* on the outcomes of the participants.

Participant heterogeneity was another limitation of these studies. The heterogeneity was mainly associated with the training level. Some of the studies [24,25,26] involved well-trained athletes with more than 5 years of experience. The remaining studies recruited individuals who exercised recreationally. Differences in skill level probably also explain the discrepancies in the exercise protocols used in individual studies. Furthermore, it is worth noting that differences in fitness levels can also influence the adaptations induced by *C. militaris*. For example, in elite athletes, the effects of supplementation with various substances are generally smaller than in recreational athletes or amateurs, as their bodies are already highly adapted due to years of intensive training.

Regarding discrepancies in exercise protocols, all studies focused solely on the aspect of endurance. Three studies [22,23,24] assessed the effect of *C. militaris* supplementation on strength, e.g., the number of repetitions performed. However, the authors of the analyzed studies did not examine the impact of *C. militaris* on lean body mass or muscle mass. This is particularly noteworthy given that the increase in lean body mass or muscle mass is the ultimate goal of using ergogenic nutrients [2].

Finally, it is worth mentioning that all the included studies have evaluated the effect of *C. militaris* supplementation for at least 1 week. None of the supplementation protocols tested the effect of acute or short-term (less than 7 days) use of *C. militaris*.

## 5. Indications for Future Studies on the Ergogenic Properties of *Cordyceps militaris* in Humans

**Figure 3:**
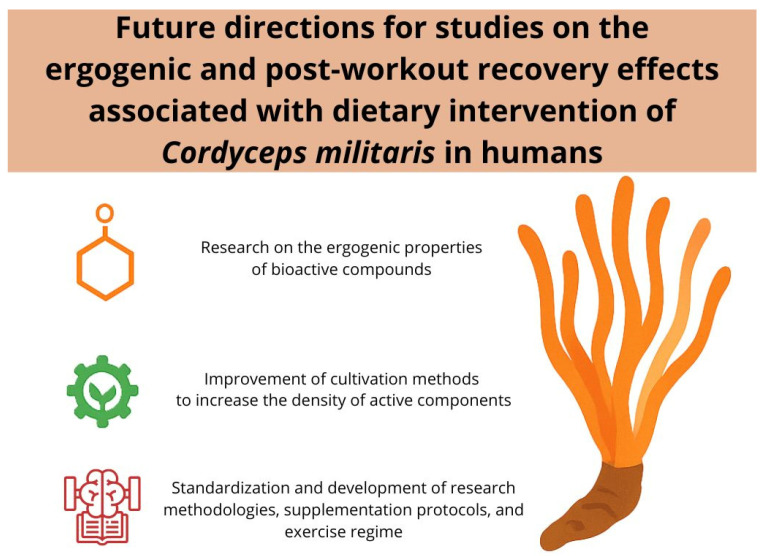
Proposals for future research directions on applying *C. militaris* supplementation in human sports and physical activity.

Screening of the scientific literature enabled us to plan for future research on using *C. militaris* supplementation in human sports and physical activity. Issues related to this topic are grouped into three categories, as presented in Figure 3.

### 5.1. Ergogenic Potential of Bioactive Compounds

A literature review on this topic indicates that *C. militaris* is a rich source of cordycepin. Two of the 16 *C. militaris* fruiting body samples contained cordycepin at levels of 325–377 mg/100 g dry weight [32]. The cordycepin content in chloroform/methanol (2:1), ethanol, and water extracts of *C. militaris* fruiting bodies was 7.25, 8.37, and 5.28 mg/g dry weight, respectively [33]. Furthermore, the cordycepin content in wines produced from solid waste cultivation and fruit wines was 7.96 and 66.78 mg/kg dry weight, respectively [34].

Cordycepin, or 3′-deoxyadenosine, is a water-insoluble organic compound belonging to the group of nucleosides [35]. It has considerable therapeutic potential and is used in various biological applications mainly because of its anticancer activity and antioxidant and anti-inflammatory effects [36]. Cordycepin shows nutritional and health-promoting potential in patients with diabetes and cardiovascular diseases and possesses immunomodulatory, anti-osteoporotic, and anti-arthritic effects, implying that this compound may be likely classified as a nutraceutical in the future [37]. A nutraceutical is a food or part of a food that not only provides health benefits but also influences the prevention and treatment of diseases [38].

**Figure 4:**
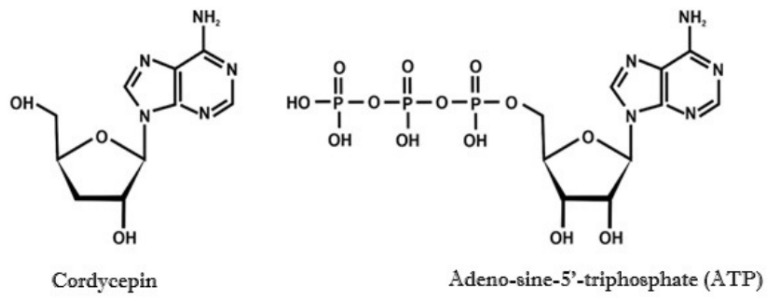
Similarities in the chemical structures of cordycepin and ATP.

The presumed ergogenic recovery properties of cordycepin are related to its potential function as an ATP precursor. However, so far, this mechanism has only been demonstrated in studies on animal models. In one study, mice were fed a diet enriched with *C. militaris* extract for 12 weeks, which was equivalent to 2.33 mg/g of cordycepin. The tested extract showed anti-inflammatory effects, with a potential reduction in the inflammatory process in response to physical exertion. These effects were achieved through interactions involving adenosine receptors, adenosine monophosphate-activated protein kinase (AMPK), and the ATP signaling pathway as well as an increase in the concentration of markers such as phosphocreatine and peroxisome proliferator-activated receptor gamma (PPAR-γ) [39]. Another study on mice also found a correlation between *C. militaris* fruiting body extract administration and anti-fatigue effects and the impact on AMPK and AKT/mTOR signaling pathways [18]. Both cordycepin and ATP share similar chemical structures (Figure 4).

Cordycepin also exhibits antioxidant and anti-inflammatory properties. Cheng et al. examined the effect of intragastric administration of cordycepin, one of the main active ingredients of *C. militaris*, in mice subjected to a high-intensity forced exercise test. All three doses of cordycepin (5, 10, and 25 mg/kg/day) increased the endurance of mice, as evidenced by a significantly higher total running time on the treadmill compared to that of the placebo group. Cordycepin also caused significant changes in biochemical parameters by increasing glycogen stores (muscle and liver) and superoxide dismutase (SOD) activity and decreasing CK, lactic acid (LA), and malondialdehyde (MDA) concentrations [31]. The results of this study were consistent with the findings of an experiment conducted by Chai et al., in which cordycepin at 20 and 40 mg/kg doses and 500 mg/kg taurine were administered to mice undergoing a swimming test for 28 days. Cordycepin increased ATP and muscle glycogen and decreased certain markers of muscle fatigue, including LA, MDA, and SOD. Metabolomics analysis showed that the anti-fatigue properties of cordycepin may be associated with the regulation of energy metabolism and the pentose phosphate pathway. The author also indicated that cordycepin participates in the activation of the TIGAR/SIRT-1/PGC-1α signaling pathway [40]. Sirtuin 1 (SIRT-1) is a type II protein deacetylase that regulates metabolic and physiological processes, including energy balance [41].

In one study, the effects of cordycepin on humans were assessed in a small group of 10 participants of both sexes. For 8 weeks, these participants were given a functional drink prepared from *C. militaris* immersion fermentation, which contained 2.85 mg of cordycepin. Following this supplementation, the female participants showed a reduced level of interleukin 6 (IL-6), while the male participants exhibited a reduction in IL-1β level. The results of this study suggest that the antioxidant and anti-inflammatory properties of cordycepin vary to some extent according to gender [42].

Studies on phytochemical composition and bioactive components show that *C. militaris* is a valuable source of ergothioneine (2-thiol-L-histidine-betaine); however, the literature indicates that the ergothioneine content varies across different *C. militaris* strains. In 2 of 16 samples, the ergothioneine content in fruiting bodies and post-harvest substrate from different strains of *C. militaris*, using different cultivation substrates, reached 26.20–29.58 mg/100 mg dry weight [32]. Other studies reported that the ergothioneine content in wild *C. militaris* was 130–140 mg/kg dry weight of mycelium and 382–799 mg/kg dry weight of fruiting bodies [33,43].

**Figure 5:**
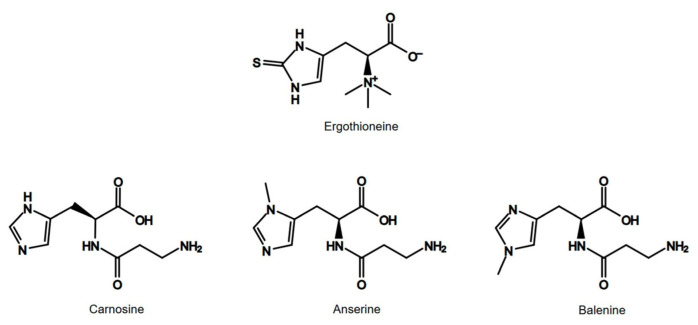
Similarities in the chemical structures of ergothioneine and imidazole dipeptides.

Ergothioneine is a sulfur derivative of L-histidine with an attached betaine fragment [44]. The presence of L-histidine shows that ergothioneine may have antioxidant properties similar to those of imidazole dipeptides: anserine, balenine, and carnosine. Histidine is an amino acid and functions as an intracellular buffering agent, particularly during anaerobic exercise. It also exhibits antioxidant properties, including chelation of metal ions and scavenging of reactive oxygen species (ROS). The antioxidant activity of ergothioneine and imidazole dipeptides is probably associated with the change in the activity of oxidative markers such as SOD, glutathione (GSH), or catalase (CAT) [45]. The similarities in the chemical structures of ergothioneine and imidazole dipeptides are shown in Figure 5.

Although ergothioneine is not biosynthesized in the human body, high concentrations of ergothioneine have been confirmed in many tissues, e.g., erythrocytes and bone marrow. An association between aging and a reduced ergothioneine level in the body has also been reported. Ergothioneine deficiency is one of the possible causes of accelerated aging and premature development of age-related diseases, e.g., dementia or frailty; however, ergothioneine concentration is not reduced during sarcopenia [46,47,48,49,50].

**Figure 6:**
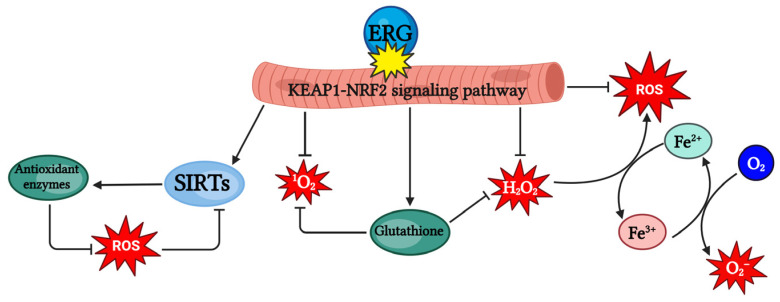
Antioxidant properties of ergothioneine.

Ergothioneine has high antioxidant potential, which demonstrates its comprehensive role in the body (Figure 6). Limiting the auto-oxidation of free copper and iron ions in cells minimizes biomolecular damage, leading to the formation of ROS. The second important aspect is the role of ergothioneine in the KEAP1-NRF2 signaling pathway. The anti-aging properties of ergothioneine could be explained through dual mechanisms. The interaction of ergothioneine with the KEAP1-NRF2 pathway increases the level of GSH redox, which further promotes the activity of the enzyme S-adenosylmethionine (SAM) synthase and increases the catalytic activity of methyltransferases. Additionally, the KEAP1-NRF2 pathway–ergothioneine interaction regulates the expression level of SIRTs. SIRT6, a histone deacetylase, influences inflammatory and oxidation processes. Moreover, animal model studies have shown that ergothioneine regulates the pathways of other antioxidant genes, e.g., SOD or heme-oxygenase-1 (HO-1), in a dose-dependent manner. The antioxidant properties of ergothioneine can thus be linked with issues related to genomic repair and stability and epigenetic modifications [51,52,53].

Previous studies on animal models, involving acute administration and several weeks of supplementation, have yielded promising results regarding ergothioneine use during increased physical activity. A daily dose of 209 ng/mg body weight of ergothioneine obtained from the diet improved all training parameters (distance, speed, and performance) in mice during 8 weeks of voluntary running on a rotating wheel [54]. Another study tested weekly ergothioneine supplementation with a daily dose of 70 mg/kg body weight. The mice underwent a TTE test on a treadmill with increasing running speed, preceded by a 10 min warm-up. Ergothioneine intervention increased the maximum aerobic speed of mice, prolonged TTE, increased protein synthesis markers, and reduced oxidative stress markers [55]. Positive results were also obtained in three additional studies, which involved a total of 50 stallions. The animals were subjected to various exercise protocols: maximum race over 2000 m, regular race over 1800 m on a standard track, and endurance training over a distance of 30 km. Ad hoc, 4-week and 2-month administration of ergothioneine in doses corresponding to 0.2, 0.5, and 0.02 mg/kg of the stallion’s body weight contributed to positive changes in several analyzed parameters, e.g., a reduction in oxidative markers, regulation of hematological indicators, and an increase in the level of the HSP-70 protein with immunomodulatory and anti-inflammatory properties [56,57,58].

Another noteworthy finding is that the only sport-related study to date on ergothioneine administration in humans failed to yield significant results. Healthy male individuals took 2.77 mg of ergothioneine from a shiitake mushroom extract (*Lentinus edodes*) for 10 days. The participants performed two independent exercise tests before a 90 min treadmill run. The only relevant finding of this study is an increase in post-exercise nitric oxide (NO) levels. Ergothioneine administration did not reduce oxidative stress markers [59]. In another study, supplementation of 5 mg or 25 mg/day of ergothioneine for 7 days in healthy volunteers showed minimal influence on changes in inflammatory biomarkers and oxidative damage [60].

As reported earlier, ergothioneine also has nootropic effects. Ergothioneine administration to elderly individuals with mild cognitive impairment yielded positive results in terms of learning ability [61], psychomotor speed, sleep initiation, and prospective memory [62,63]. The nootropic effects of ergothioneine are likely related to its interaction with tropomyosin receptor kinase B (TrkB). TrkB plays a crucial role in promoting neuroplasticity for proper learning and memory. The impact of ergothioneine deficiency in the daily diet reduced cognitive function and decreased hippocampal neurogenesis in mice. Thus, the role of ergothioneine in regulating TrkB phosphorylation levels in the hippocampus and extracellular vesicles may explain its nootropic properties in humans [64].

Both cordycepin and ergothioneine require further study, as their potential ergogenic and pro-recovery mechanisms have only been documented in animal model studies.

### 5.2. Role of Cultivation Methods on the Content of Active Ingredients in C. militaris

Genome editing and sustainable biotechnology are key to scaling up *C. militaris production*. Genetic engineering supports stable growth, improved resistance, efficient use of agro-waste, and enhanced biosynthesis of secondary metabolites such as cordycepin, ergothioneine, and polysaccharides [65]. Techniques like CRISPR/Cas9 enable targeted pathway optimization, leading to significantly increased metabolite yields [66,67]. New high-yielding strains can be developed through hybridization, cross-breeding, and mutagenesis, resulting in enhanced cordycepin production and broader pharmaceutical and food application [68,69,70].

Metabolite content can also be increased by optimizing culture media and supplementation with selected nutrients, plant additives, trace elements, or bioactive compounds, which substantially improved cordycepin levels [71,72,73,74,75,76,77,78,79,80]. Additionally, fermentation strategies and environmental factors–such as pH, temperature, light, and oxygen availability–strongly influence metabolite biosynthesis [81,82,83].

Although insect-based substrates promote high metabolite production, they are impractical for large-scale industry due to complexity and cost [84,85,86]. Therefore, plant-based substrates are preferred for commercial cultivation, despite ongoing challenges related to the fungus’s entomopathogenic nature and specific physiological requirements [85,87].

Park [81] reported that many limitations still need to be overcome to achieve reproducible and consistent large-scale cultivation of *C. militaris*. Among the main limitations, the author cited the lack of standardized cultivation protocols, significant variability in production systems, and economic and engineering constraints. These latter issues, such as inefficient oxygen transfer in bioreactors, poor foam management during aerated fermentation, and ineffective strategies for further metabolite purification, must be addressed before considering full-scale industrial production.

### 5.3. Standardization and Development of Research Methodology

Future studies should aim to address the limitations mentioned in Table 1 and Table 2. It is crucial to use mushroom materials standardized to achieve a specific content of certain active ingredients, including cordycepin and ergothioneine. The dose size should also be standardized for the optimal production of mushroom products and the amount of a specific bioactive component. For example, previous studies on the effects of ergothioneine on physical activity have used doses considerably lower than the current limit set by EFSA (470 mg/kg body weight) [88].

Future studies should also focus more on acute supplementation or supplementation for less than 7 days. *C. militaris* supplementation for improving physical activity should cover two aspects: ergogenic effects and support for post-exercise recovery. Firstly, components with a well-documented effect, classified as group A in the AIS classification, should be considered, including beta-alanine, glycerol, nitrates, caffeine, creatine, and sodium bicarbonate [89,90].

Based on our literature review, a few other, less obvious directions may be worth exploring. The cordycepin content of the freeze-dried and powdered mycelium doubled by adding 8 g/L L-alanine to *C. militaris* culture on potato dextrose agar medium [91]. Furthermore, L-alanine addition at 12 g/L dose induced a three-fold increase in cordycepin content compared to that in the control culture [92]. However, the use of L-alanine as a supplement to endure physical activity remains to be fully validated. As reported previously, the administration of a carbohydrate-protein solution or L-alanine to cyclists did not provide benefits, but may rather have a negative effect on their physical performance [93]. Additionally, alanine administration before and during the exercise test had a beneficial effect on improving metabolic parameters but did not alter physical performance [94]. Another worthwhile study on L-alanine supplementation is the study of Ueda et al., in which this amino acid was administered along with L-arginine and L-phenylalanine. The administration of a 3 g mixture of these three amino acids in combination with low-intensity exercise was beneficial for ketone body synthesis and increased adipose tissue recruitment [95,96,97]. Therefore, it would be interesting to investigate the simultaneous use of *C. militaris* standardized for cordycepin content along with the aforementioned amino acids. This might enable assessment, for example, of the role of cordycepin in the ketogenesis process. Studies on animal models, wherein a mixture of L-alanine with BCAA amino acids or L-glutamine was administered to mice and rats, reported optimal results related to enhanced protective effects on muscles subjected to intense exercise [98,99].

Ergothioneine supplementation should certainly be considered along with other imidazole dipeptides, namely anserine, balenine, and carnosine, and also with other antioxidant compounds. A good example is N-acetyl-L-cysteine (NAC), whose supplementation in physically active individuals shows beneficial effects, for example, in reducing muscle soreness [100] or improving performance capacity [101]. It would be intriguing to examine the combination of these two components, given that the only study to date evaluating the effect of simultaneous administration of NAC and ergothioneine is an in vitro experiment, which showed that supplementation with this combination had no effect on the change in skeletal muscle contraction speed during exercise testing [102].

Ingredients such as ergogenic pycnogenol [103] and omega-3 fatty acids should also be further investigated, as they have been shown to possess valuable anti-inflammatory properties and reduce the negative effects of intense physical activity, such as delayed muscle soreness or oxidative stress-induced microdamage [104].

Future studies should also assess the form of *C. militaris* supplementation. For example, researchers should closely examine the use of liposomal technology for *C. militaris*-based preparations as a source of active ingredients. A recently developed nanoliposome modified with chitosan oligosaccharides improved cordycepin stability, protected it from rapid degradation, and enhanced its antioxidant activity [105]. In recent years, medicinal mushrooms have been extensively studied for application in the pharmaceutical, food, and sports industries; hence, *C. militaris*-derived materials can serve as a starting point for creating products and supplements that can complement the daily diet. Functional drinks, energy shots, and teas can be a source of both *C. militaris* alone and carefully selected mushroom blends [106]. Superfine grinding of *C. militaris* fruiting bodies improved the efficiency of cordycepin production and increased the ability to scavenge free radicals [107]. Traditionally brewed coffee supplemented with *C. militaris* fruiting bodies is a very good source of many valuable elements such as zinc, magnesium, copper, potassium, sodium, and calcium. The presence of fruiting bodies also increased the content of 4-feruloylquinic acid and 3,5-dicaffeoylquinic acid [108]. The combination of *C. militaris* with peri-exercise supplementation with fruit and vegetable juices, which are widely used in sports nutrition, should also be considered. For example, *C. militaris* could be combined with beet juice, a source of ergogenic nitrates; watermelon juice, a source of L-citrulline; and cherry and pomegranate juices, which are rich in polyphenols that support tissue regeneration [109].

Future studies should also ensure homogeneity of the participants. Age, sex, gender, body weight, fitness level, and type of sport practiced should be as similar as possible to minimize discrepancies in the results. Furthermore, additional parameters should be considered to evaluate the application of *C. militaris* and its active ingredients. For this purpose, it would be worthwhile to choose proven exercise protocols whose effectiveness has been measured and validated. For example, the 1RM test [110] or a handheld dynamometer [111] could be used to assess changes in muscle strength.

It also seems reasonable to examine the parameters and use tools, protocols, and tests recommended for specific sports disciplines. For example, the maximum counter-movement jump (CMJ) could be used to assess improvements in basketball players [112], while a tennis agility test (TAT) might be appropriate for tennis players [113].

### 5.4. Additional Findings

The assessment of the role of *C. militaris* in post-exercise recovery should not be limited to issues solely related to training and muscle fatigue. Structural damage to the musculoskeletal system commonly occurs in physically active people. It ranges from fractures, dislocations, and displacements to inflammatory changes in tendons and leads to the formation of trigger points or painful overload syndromes such as shin splints [114]. A study of 321 participants of both sexes, most of whom practiced yoga, capoeira, and running, showed that as many as 78.4% of the respondents had experienced chronic wrist pain at least once. The study revealed an interesting, though statistically insignificant, trend: The incidence of chronic pain was highest among the youngest participants (18–25 years) at 63.8% [115]. A systematic review of seven studies involving 346 adult male and female athletes showed that more than 50% of the athletes treated pain with oral or topical anti-inflammatory drugs. The authors also indicated that nonsteroidal anti-inflammatory drugs (NSAIDs) are effective only for acute pain, and the efficacy of these drugs decreases in cases of chronic injuries or pain syndromes [116]. In another study, athletes keenly used pain relief products. Of 731 young elite Danish athletes (aged 15–20 years), 31% of athletes confirmed that they had used painkillers at least once in the last four weeks. The authors noted that the probability of using painkillers increased with the intensity of pain [117]. More than half of the 230 American female athletes and almost one third of the 83 male athletes suffered from various pain conditions during 8 weeks of training. One in four female athletes and one in five male athletes confirmed the use of NSAIDs to relieve pain. Most study participants purchased their medication themselves [118]. However, it remains to be confirmed whether the use of NSAIDs has an ergogenic effect, e.g., improving performance. There are also real health-related risks associated with the use of these drugs, including electrolyte imbalance, hypernatremia, oxidative stress, inflammatory changes and muscle damage, acute kidney injury, and gastrointestinal disorders [116,119,120].

Based on its bioactive components and health-beneficial properties, *C. militaris* can be categorized as a supplementary component of the daily diet for physically active people, which possibly allows them to maintain their musculoskeletal system [37]. NSAIDs and *C. militaris* have an almost similar anti-inflammatory potential. Both of them can reduce ROS production, nuclear factor kappa-light-chain-enhancer of activated B cells (NF-κB) activation, arachidonic acid metabolite production, and proinflammatory cytokine release. These reactions are specific components of the inflammatory process [121]. Therefore, it would be interesting to investigate the combination of *C. militaris* supplementation with other natural ingredients proven to be beneficial in alleviating pain, such as ginger [122], *Boswellia serrata* [123], or turmeric [124]. *C. militaris* should also be explored as an element of diet and supplementation for providing structural support to the musculoskeletal system. In vitro and in vivo studies on animal models suggest that cordycepin may prevent bone loss, promote osteogenesis, and accelerate fracture healing. *C. militaris* mushrooms are also a rich source of calcium, phosphorus, and vitamin D, which are components of bone mineral mass [125].

The NIH label database includes 733 labels (<1% of the database) for the keyword “cordyceps.” This finding indicates that *Cordyceps* spp. are not yet a common ingredient in the food industry, contrary to their popularity in society [126]. There have been many unfortunate cases of adulteration or contamination of dietary supplements or functional foods with unauthorized ingredients, such as the case of using anabolic-androgenic steroids (AAS) instead of *Tribulus terrestris* and contamination of phenethylamines (PEAs) or aliphatic amines associated with various botanical extracts [127,128]. The ongoing intensive studies on *O. sinensis* and *C. militaris* have demonstrated the value of these mushrooms as a nutritional/food supplement and a source of many bioactive compounds, including nucleosides, amino acids, polyamines, and peptides, thus confirming their medicinal/therapeutic potential. In the context of food adulteration or contamination, the cultivation and distribution of these mushrooms should be regulated and monitored based on additional guidelines. According to the Act Governing Food Safety and Sanitation (Food and Drug Administration, Ministry of Health and Welfare, Taiwan), the conditions of *C. militaris* manufacture and usage as a food supplement are proposed to be restricted; moreover, food products containing *C. militaris* are required to be labeled accordingly with warning information. The primary objective and rationale for this approach are “prevention of deceptive practices and protection of consumer health” [129].

An analysis of 50 dietary supplements from the testosterone booster (TB) category available in 10 sales markets conducted in 2022 showed that 10% of the products contained *C. militaris* or *O. sinensis* [130]. *C. militaris* is likely used in dietary supplements classified as TB because of its impact on hormonal balance. In a group of 66 patients with confirmed bladder issues (e.g., weak stream, difficulty in urinating, and excessively frequent urination), some participants received *C. militaris* capsules (equivalent to 24 g of cordycepin, 0.36 g of adenosine, and 40 g of polysaccharides) for 92 days. The administration of *C. militaris* fruiting bodies possibly contributed to reducing bladder obstruction and decreasing prostate size [131]. The administration of *C. militaris* fruiting body extract to mice enabled the maintenance of testosterone and dihydrotestosterone (DHT) levels and increased their secretion. *C. militaris* inhibited testosterone-induced prostate hyperplasia [132]. In another study, 30 male rats were administered either a placebo or *C. militaris* extract at doses of 50 or 200 mg/kg body weight. The animals consumed the mushroom material for 30 days and were simultaneously exposed to the negative effects of tobacco smoke. Rats consuming *C. militaris* showed a decline in testosterone levels and the number of Leydig cells [133]. Cordycepin extracted from *C. militaris* mycelium was administered to rats for 6 months at doses of 5, 10, and 20 g per day. The animals receiving cordycepin showed increased motility, progression, and average speed of spermatozoa. However, the maximum dose of 20 g/day mitigated changes in the expression of proteins and mRNA associated with the spermatogenesis process [134]. Considering *C. militaris* as a potential testosterone/hormone booster opens several new avenues for research on its similarities and differences with other ingredients used as sport supplements. Apart from those ingredients with a large amount of scientific evidence or EU regulatory documents, such as *T. terrestris*, *Panax ginseng*, or L-arginine [130], there are several other natural substances that are equally interesting based on their effects and applications, such as phytoecdysteroids like ecdysterone [135], *Eurycoma longifolia* (longjack/tongkat ali) [136] and *Trigonella foenum-graecum* (fenugreek) [137,138].

*C. militaris* can also be found among the ingredients of products marketed as “neurogenic aids” supplements, although this term functions predominantly as a marketing construct rather than a scientifically grounded category of substances with demonstrable cognitive effects [139].

*C. militaris* is also a rich source of L-phenylalanine, with content exceeding 500 mg/100 g dry weight in fruiting bodies [32]. L-phenylalanine is an essential aromatic amino acid supplied with the daily diet. Phenylalanine plays the primary role of a tyrosine precursor, an essential component for synthesizing critical neurotransmitters such as dopamine, epinephrine, and noradrenaline [140,141,142].

To date, the available literature provides only limited insight into the independent use of phenylalanine in humans for improving physical activity. A 3 g serving of phenylalanine administered to actively exercising men approximately 30 min before exercise on a bicycle ergometer increased plasma glycerol and glucagon concentrations in the participants. These changes probably indicate that phenylalanine promotes fat oxidation [143]. Further studies are warranted to clarify the role of phenylalanine in the ergogenic properties of *C. militaris*.

## 6. Conclusions

The findings of this narrative review indicate that *C. militaris* supplementation may provide beneficial ergogenic and post-exercise recovery effects in physically active individuals, including improvements in VO_2max_, TTE, and power output and also in maintaining SpO_2_ levels during high-intensity exercise (reported in one study). Several studies have also shown that *C. militaris* supplementation induced favorable changes in selected biochemical markers associated with muscle damage and inflammation, such as CK, BUN, and WBC. However, the overall certainty of these observations is limited because of moderate to high risk of bias; absence of standardized preparations of fungal material; and substantial methodological heterogeneity across supplementation protocols, exercise tests, and participant characteristics.

In terms of practical application, the current evidence is insufficient to support definitive recommendations regarding the use of *C. militaris* in sports nutrition. Although preliminary findings suggest potential benefits for endurance performance and post-exercise performance, the small number of available studies, their methodological limitations, and the variability in supplementation protocols and outcome measures limit the strength and generalizability of these observations. Consequently, the use of *C. militaris* in physically active populations should be regarded as investigational rather than evidence-based at this stage.

Future research should aim to address these limitations through rigorously designed randomized controlled trials employing chemically standardized preparations with defined bioactive compound profiles, clearly characterized athletic populations, and validated, sport-specific performance outcomes. Additional investigations exploring dose–response relationships, acute supplementation strategies, strength-related parameters, and comparisons with established ergogenic aids would contribute to a more comprehensive understanding of the physiological relevance, safety, and practical applicability of *C. militaris* supplementation in sport.

## Figures and Tables

**Table 1:** Characteristics of the studies included in the narrative review.

Reference	Study Design	Participant Characteristic	Mean Age, Years (±SD)	Duration of Supplementation (Weeks)	Mushroom Material	Dose	Exercise Protocols	Investigated Parameters	Outcomes Measured				Results	Outcome	Safety Assessment	Full Composition/Concomitant Ingredients Per Single Dose	Funding
									Baseline CMG	Post CMG	Baseline PL	Post PL					
Hirsch et al. (2017) [22]	Double-blinded, placebo-controlled	Healthy recreationally active individuals of both sexes *n* = 28 M = 16 F = 12	22.7 ± 4.1	1	*C. militaris* (as a part of dietary supplement PeakO_2_^®^)	3 × 2 caps/day (4 g *C. militaris*/day)	Three-minute maximal cycle test	VO_2max_ (ml/kg/min)	47.7 ± 9.4	49.0 ± 8.6	46.4 ± 7.9	**48.9 ± 8.1**	↑ both groups stronger ↑ PL	Three weeks of supplementation proved to be more valuable for participants	No information	PeakO_2_^®^ is an organic combination of six Ayurvedic mushroom strains: *C. militaris*, *G. lucidum*, *Pleurotus eryngii, L. edodes, Hericium erinaceus*, and *Trametes versicolor* Content of any bioactive compounds not determined	Disruptive Nutrition LLC, North Carolina Biotech Center
								VT (L/min)	N/D	N/D	N/D	N/D	↔ in both groups				
								TTE (s)	855.1 ± 135.9	**883.2 ± 132.7**	818.2 ± 250.0	876.1 ± 169.7	↑ CMG ↔ PL				
								RPP (W∙kg^−1^)	5.9 ± 1.0	6.3 ± 1.8	6.0 ± 0.8	5.9 ± 1.0	↔ in both groups				
								AvgP	N/D	N/D	N/D	N/D	↔ in both groups				
								%drop	N/D	N/D	N/D	N/D	↔ in both groups				
		Healthy recreationally active individuals of both sexes *n* = 10 M = 4 F = 6	21.7 ± 2.7	3	*C. militaris* (as a part of dietary supplement PeakO_2_^®^)	3 × 2 caps/day (4 g *C. militaris*/day)	Three-minute maximal cycle test	VO_2max_ (mL/kg/min)	44.0 ± 10.5	**48.8± 11.2**	45.0 ± 12.5	45.9 ± 9.9	↑ CMG ↔ PL				
								VT (L/min)	11.7 ± 0.3	**2.4 ± 1.0**	2.4 ± 0.9	2.4 ± 0.7	↑ CMG ↔ PL				
								TTE (s)	851.7 ± 192.2	**921.5 ± 184.2**	880.0 ± 204.7	884.6 ± 147.5	↑ CMG ↔ PL				
								RPP (W∙kg^−1^)	5.9 ± 1.1	**6.4 ± 1.8**	5.7 ± 0.9	5.7 ± 0.5	↑ CMG ↔ PL				
								AvgP	N/D	N/D	N/D	N/D	↔ in both groups				
								%drop	72.0 ± 6.3	70.7 ± 8.1	76.1 ± 3.9	**71.3 ± 6.7**	↓ both groups stronger ↓ PL				
Dudgeon (2018) [23]	Randomized, single blinded, placebo-controlled	Healthy recreationally active individuals of both sexes *n* = 43 M = 21 F = 22	22.0 ± 2.8	4	*C. militaris* (as a part of dietary supplement PeakO_2_^®^) Placebo group	1–2 g/day PeakO2^®^ + 2 g of Gatorade	VO_2peak_ incremental exercise test on cycle ergometer Wingate anaerobic power test	TTE (s)	N/D	**25.8 ± 61.1**	N/D	0.5 ± 30.5	↑ CMG ↔ PL	Four-week supplementation with *C. militaris* positively affected all measured parameters compared to placebo One-week supplementation with *C. militaris* benefited mainly less-trained participants; other outcomes were variable	One subject suffered from adverse response of gastrointestinal distress; one subject suffered from severe quadriceps DOMS	PeakO_2_^®^ is an organic combination of six Ayurvedic mushroom strains: *C. militaris*, *G. lucidum*, *Pleurotus eryngii, L. edodes, Hericium erinaceus*, and *Trametes versicolor* Content of any bioactive compounds not determined	Disruptive Nutrition LLC, North Carolina Biotech Center
								BL (mM)	N/D	**0.22 ± 0.41**	N/D	0.01 ± 0.52	↓ CMG ↔ PL				
								VO_2peak_ (mL/kg/min)	40.1± 6.1	**41.1 ± 6.2**	401.7 ± 6.1	42.5 ± 6.0	↑ CMG ↔ PL				
		Healthy recreationally active individuals of both sexes *n* = 40 M = 24 F = 16	23.1 ± 4.9	1	*C. militaris* (as a part of dietary supplement PeakO_2_^®^) Placebo group	12 g/day PeakO_2_^®^ +12 g Gatorade	VO_2peak_ incremental exercise test on cycle ergometer Wingate anaerobic power test	TTE Top (s)	829.4 ± 118.2	852.6 ± 116.5	845.2 ± 95.3	870.7 ± 100.5	↑ in both groups				
								TTE Bottom (s)	785.5 ± 115.3	787.5 ± 97.7	823.3 ± 127.6	833 ± 174.7	↔ in both groups				
								VO_2peak_ Top (mL/kg/min)	45.4 ± 3.8	44.9 ± 4.2	46.8 ± 3.0	47.3 ± 3.0	↔ in both groups				
								VO_2peak_ Bottom (ml/kg/min)	36.9 ± 3.8	**38.9 ± 4.4**	37.5 ± 4.1	37.3 ± 5.2	↑ CMG ↔ PL				
								PPO Top (W)	932.7 ± 235.5	947.9 ± 197.0	907.5 ± 181.5	**949.7 ± 209.4**	↔ CMG ↑ PL				
								PPO Bottom (W)	919.9 ± 238.9	944.6 ± 224.6	945.3 ± 253.9	966.5 ± 285.4	↔ in both groups				
Wang (2021) [24]	No information about blinding	Healthy young swimmers *n* = 180 M = 120 F = 60	17.1 ± 1.4	7	*C. militaris*	8 g/day	30 s maximal effort cycle test 8 h of training per day divided into four sessions, 6 days a week for 7 weeks	P_max_ (W)	N/D	**1137.32 ± 78.19**	N/D	851.72 ± 140.51	↑ CMG vs. PL	Seven-week supplementation with *C. militaris* positively affected all measured parameters compared to placebo, except BL	No information	Content of any bioactive compounds not determined	No information about funding
								MP (W)	N/D	**715.21 ± 51.47**	N/D	543.91 ± 93.10	↑ CMG vs. PL				
								BL (mol/L)	N/D	13.07 ± 0.53	N/D	11.93 ± 0.53	↔ CM vs. PL				
								WBC	8.24 ± 1.33	**7.11 ± 0.37**	8.77 ± 0.46	5.48 ± 0.92	↓ both groups stronger ↓ CMG				
								Hb	151.26 ± 4.89	**131.72 ± 2.27**	148.50 ± 4.66	115.50 ± 4.13	↓ both groups milder ↓ CMG				
								CK (U∙L) *	675.41	**590.16**	655.74	708.20	↓ CMG ↑ PL				
								BUN (mmol∙L) *	6.88	**9.46**	6.40	12.54	↑ both groups milder ↑ CMG				
								T serum (µg∙L) *	56.77	**42.58**	57.42	22.58	↓ both groups milder ↓ CMG				
								IL-4	82.16 ± 7.16	**78.47 ± 2.07**	84.38 ± 2.77	82.46 ± 2.77	↓ CMG ↔ PL				
								IFN-γ	879.47 ± 46.13	**960.39 ± 46.13**	990.24 ± 8.27	1006.92 ± 8.18	↑ both groups stronger ↑ CMG				
Nakamura (2024) [25]	Double-blind, placebo controlled	Healthy male long-distance runners *n* = 22 M = 22 F = 0	18–24	16	*C. militaris*	6 capsules/day (1.8 g *C. militaris*/day)	Assessment of participants during preseason training sessions	Total distance run (km)	N/D	1623.5 ± 389.1	N/D	1732.0 ± 231.9	↔ CMG vs. PL	The applied supplementation proved beneficial only in lowering WBC and CK levels.	No information	1 capsule contains 300 mg *C. militaris* Content of any bioactive compounds not determined	La Vie En Sante Ltd. Ibaraki, Japan
								Running time over 5000 m (min)	15:22.75 ± 00:35.31	14:55.98 ± 00:33.93	15:18.82 ± 00:31.60	14:54.44 ± 00:32.00	↓ both groups ↔ CMG vs. PL				
								EPO (mlU/mL)	6.98 ± 2.14	10.55 ± 2.96	6.75 ± 1.74	9.16 ± 1.98	↑ both groups ↔ CMG vs. PL				
								WBC (/mL)	5627 ± 716	**5236 ± 452**	7127 ± 1861	5918 ± 643	↓ both groups stronger ↓ in CMG				
								CK (U/L)	336.73 ± 249.79	**225.82 ± 109.49**	427.91 ± 399.60	384.64 ± 188.84	↓ both groups stronger ↓ in CMG				
								GT (IU/L)	21.45 ± 5.65	21.09 ± 5.99	22.73 ± 5.35	22.00 ± 5.50	↔ between and both groups				
								C-reactive protein (mg/dL)	0.05 ± 0.03	0.06 ± 0.03	0.36 ± 0.89	0.05 ± 0.02	↔ between and both groups				
								BUN (mg/dL)	18.92 ± 6.37	15.21 ± 4.78	19.85 ± 5.93	15.62 ± 3.41	↓ both groups ↔ CMG vs. PL				
								UA (mg/dL)	5.85 ± 0.89	4.94 ± 0.78	5.78 ± 0.80	**4.95 ± 0.66**	↓ both groups stronger ↓ in PL				
								CREAT (mg/dL)	0.82 ± 0.06	0.80 ± 0.05	0.78 ± 0.08	0.78 ± 0.08	↔ between and both groups				
								T (ng/mL/L)	7.24 ± 2.39	7.01 ± 1.76	6.25 ± 1.85	6.09 ± 1.95	↔ between and both groups				
Pasha et al. (2024) [26]	Double-blind, placebo controlled	Healthy young runners of both sexes *n* = 48 M = 24 F = 24	16–35	3	*C. militaris* Placebo group	1 g/day	Time test for treadmill Running for distances: 200 m and 5 km	SpO_2_ (%)	N/D	**95 ± 1.5**	N/D	72 ± 1	↑ CMG vs. PL	Post-intervention values suggest improvements in all measured parameters. Baseline is not reported; therefore, within-group changes and formal comparisons cannot be assessed.	No information	Content of any bioactive compounds not determined	No external funding
								Treadmill TT (min)	N/D	**5 ± 1**	N/D	2 ± 3	Ability to complete the test in CMG Incomplete test in PL				
								Running time over 200 m (s)	N/D	**25 ± 2**	N/D	40 ± 1	↓ CMG vs. PL				
								Running time over 5 km (min)	N/D	**13.5 ± 1**	N/D	18 ± 1.4	↓ CMG vs. PL				
					*C. militaris* + whey protein Placebo group	1 g + 10 g/day	Time test for treadmill Running for distances: 200 m and 5 km	SpO_2_ (%)	N/D	**93 ± 1.2**	N/D	68 ± 1.8	↑ CMG vs. PL				
								Treadmill TT (min)	N/D	**4 ± 1.1**	N/D	2.2 ± 0.5	Ability to complete the test in CM Incomplete test in PL				
								Running time over 200 m (s)	N/D	**28 ± 0.5**	N/D	35 ± 1	↓ CMG vs. PL				
								Running time over 5 km (min)	N/D	**14.7 ± 1.8**	N/D	17 ± 1.2	↓ CMG vs. PL				

Abbreviations: **bold**: statistically significant result; ↑: significant increase in the value of the investigated parameter; ↓: decrease in the value of the investigated parameter; ↔: nonsignificant changes in the value of the investigated parameter; AvgP: average power; BL: blood lactate; BUN: blood urea nitrogen; CK: creatine kinase; CMG: *Cordyceps militaris* group; CREAT: creatinine; EPO: erythropoietin; IFN-γ: interferon gamma; IL-4: interleukin 4; Hb: hemoglobin; MP: mean power; N/D: not determined; PL: placebo group; P_max_: maximal power output; PPO: peak power output; RPP: relative peak power; SpO_2_: peripheral oxygen saturation; StO_2_: muscle tissue oxygen saturation; T: testosterone; TTE: time to exhaustion; TT: time trial; UA: uric acid; VO_2max_: maximal oxygen uptake; VO_2peak_: peak oxygen uptake; VT: ventilatory threshold; WBC: white blood cells. * The values for the parameters are approximate results obtained by digitizing data from graphs using WebPlotDigitizer 5.2 software.

**Table 2:** Qualitative assessment of methodological quality and key limitations of the included studies.

References	Bias Due to the Randomization Process	Bias Due to Deviations from Intended Interventions	Bias Due to Missing Outcome Data	Bias in Measurement of the Outcome	Bias in Selection of the Reported Result	Overall Bias
Hirsch et al. (2017) [22]	**LOW**	**LOW**	MODERATE	**LOW**	MODERATE	MODERATE
Dudgeon (2018) [23]	MODERATE	MODERATE	MODERATE	**LOW**	**HIGH**	**HIGH**
Wang (2021) [24]	MODERATE	MODERATE	**LOW**	MODERATE	**HIGH**	**HIGH**
Nakamura (2024) [25]	**LOW**	**LOW**	MODERATE	**LOW**	**HIGH**	**HIGH**
Pasha et al. (2024) [26]	**LOW**	**LOW**	**LOW**	**LOW**	**HIGH**	**HIGH**

## Data Availability

No new data were created or analyzed in this study. Data sharing is not applicable to this article.

## Supplementary Materials

The following supporting information can be downloaded at: https://www.mdpi.com/article/10.3390/nu18050781/s1, File S1: Risk of Bias 2 analysis.
