# Supplementary material for: Current Evidence of Ergogenic and Post-Exercise Recovery Effects of Dietary Supplementation with Cordyceps militaris in Humans—A Narrative Review
Source: Nutrients. 2026 Feb 27;18(5):781. doi: 10.3390/nu18050781 (PMC12986667; doi:10.3390/nu18050781)
Supplement: Supplementary file 1 [file nutrients-18-00781-s001.zip › nutrients-4160048-supplementary.pdf]

Hirsch, K.R.; Smith-Ryan, A.E.; Roelofs, E.J.; Trexler, E.T.; Mock, M.G. *Cordyceps militaris* Improves Tolerance to High-Intensity Exercise After Acute and Chronic Supplementation. *J. Diet. Suppl.* 2017, *14*, 42–53. https://doi.org/10.1080/19390211.2016.1203386.

Domain	Risk of Bias	Details
Bias due to the randomization process	LOW	This study used block randomization with Random Allocation Software, and the use of unlabeled containers (placebo versus supplement) served as a form of concealment of allocation.
Bias due to deviations from intended interventions	LOW	The study was double-blind, which means that neither the participants nor the researchers knew the group assignments. Compliance with the study protocol was monitored (≥ 90% compliance).
Bias due to missing outcome data	MODERATE	Although most participants completed the study, there were some limitations. Two people did not complete phase I, and only 10 participants took part in phase II. It is therefore possible that the lack of sufficient data affected the results of the second stage of the study.
Bias in measurement of the outcome	LOW	The authors of the study used objective performance parameters (VO_2max, TTE, VT) measured during an exercise protocol on a cycle ergometer. The individuals responsible for the measurements were blinded to the intervention.
Bias in selection of the reported result	MODERATE	Although the study was registered on ClinicalTrial.gov (NCT02075892) and the reported results reflect the methods described, there is a risk of omitting secondary outcomes.

Final score:

Moderate risk of bias: This study is characterized by a solid methodological design, as evidenced by randomization, double blinding, and the use of objective measures. Unfortunately, minor data gaps and partial reporting of the second phase are the main sources of uncertainty.

David Dudgeon, W. The Effects of High and Low-Dose Cordyceps Militaris-Containing Mushroom Blend Supplementation After Seven and Twenty-Eight Days. American Journal of Sports Science 2018, 6, 1, doi:10.11648/j.ajss.20180601.11. 24.

Domain	Risk of Bias	Details
Bias due to the randomization process	MODERATE	The research method described indicates that participants were randomly assigned to groups and trials. However, the authors do not provide details on, among other things, the method of randomization, concealment of assignment, or balance between groups at the start of the study.
Bias due to deviations from intended interventions	MODERATE	The study was described as a "single-blind," but it was not specified who exactly was blinded—the participants or the researchers conducting the measurements. Compliance with the protocol was monitored, but there is no detailed description of the blinding of the personnel responsible for this issue.
Bias due to missing outcome data	MODERATE	There were 43 participants in Trial 2, but 3 of them withdrew. The authors did not describe how they handled missing data or how it might have affected the final results of the study.
Bias in measurement of the outcome	**LOW**	The study focused on objective physiological measures (VO_2peak, TTE, blood lactate concentration), which were measured using standardized diagnostic equipment. The authors mention that the persons responsible for the measurements were blinded.
Bias in selection of the reported result	**HIGH**	This study has not been registered, as evidenced by the lack of a registration number in ClinicalTrials.gov and other databases. This means that the completeness of the reporting of results cannot be verified.

Final score:

× **High risk of bias:** Although the study has valuable methodological strengths, such as objective physiological measures and assurance of randomization, it lacks key elements of transparency, such as limited information on blinding, unclear randomization methods, and no registration.

Wang, R. Effects of Cordyceps Militaris Effective Substances on the Physical Function of Athletes. Journal of Food Safety and Quality 2021, 12, 2802–2806.

Domain	Risk of Bias	Details
Bias due to the randomization process	MODERATE	The author mentions random assignment of participants, which shows that the initial characteristics of the groups are similar. However, there is no description of the randomization method, i.e., no detailed information on sequence generation or allocation concealment.
Bias due to deviations from intended interventions	MODERATE	The study does not indicate any systematic deviations, but the author does not declare the blinding of the sample and does not mention the control of compliance with the supplementation protocol. Also missing is data on who and how supervised the supplementation protocol.
Bias due to missing outcome data	**LOW**	The data presented in the results tables are complete and include all 180 participants. Furthermore, the author does not mention any participants dropping out during the study, which eliminates any possible data gaps.
Bias in measurement of the outcome	MODERATE	Although the measured indicators are objective, there is a lack of information about random assignment and blinding of those responsible for the measurement process.
Bias in selection of the reported result	**HIGH**	This study has not been registered, as evidenced by the lack of a registration number in ClinicalTrials.gov and other databases. This means that the completeness of the reporting of results cannot be verified.

Final score:

× **High risk of bias:** Although the study has numerous participants and a sound control process design, there is a significant risk of systematic error in the results obtained. This is due to the lack of clear information on randomization, blinding, and registration. Effect of the administration of

Nakamura, A.; Shinozaki, E.; Suzuki, Y.; Santa, K.; Kumazawa, Y.; Kobayashi, F.; Nagaoka, I.; Koikawa, N. Effect of the Administration of Cordyceps Militaris Mycelium Extract on Blood Markers for Anemia in Long-Distance Runners. Nutrients 2024, 16, 1835, doi:10.3390/nu16121835

Domain	Risk of Bias	Details
Bias due to the randomization process	LOW	The authors of this study indicate that participants were assigned to groups to minimize significant baseline differences (age, Hb and EPO concentrations, best seasonal result). Furthermore, the study is a "randomized controlled trial with parallel groups" and used outcome balancing. This suggests that the randomization and allocation procedures were valid.
Bias due to deviations from intended interventions	LOW	The study was declared double-blind, involving both participants and researchers. Compliance with the recommendations was monitored at an excellent level (>99%). Any possible deviations were controlled and documented.
Bias due to missing outcome data	MODERATE	Two participants did not take part in the 5000-meter run, resulting in missing data for this particular outcome. However, for most of the other measurements, data from all 22 participants were included.
Bias in measurement of the outcome	LOW	The parameters used were objective (hematological blood markers, running time), and their measurement was carried out according to standardized designs. In turn, double-blinding reduced the risk of bias, among other things, because the individuals responsible for conducting the measurements were not aware of the assignment.
Bias in selection of the reported result	HIGH	This publication does not provide information on trial registration (no number in databases such as ClinicalTrials.gov or UMIN) or the research protocol.

Final score:

× **High risk of bias:** Despite its extremely robust methodological design, this study has two significant shortcomings: missing data for one important outcome and a lack of transparency in the reporting procedure due to non-registration. These shortcomings raise significant concerns in the context of selective reporting and the impact of missing data on the study results.

Pasha, S.M.; Rajan, A.N.; Rathod, L.; Musfera, S.; Pasha, C. Improved Oxygen Saturation and Performance of Athletes Using Cordyceps Militaris. Asian Journal of Biological Sciences 2024, 17, 85–92, doi:10.3923/ajbs.2024.85.92.

Domain	Risk of Bias	Details
Bias due to the randomization process	LOW	The authors of the study indicated that participants were randomly assigned to four groups using Random Allocation Software and received coded infusions. Baseline data were collected and no baseline imbalances were noted.
Bias due to deviations from intended interventions	LOW	This study was a double-blind trial. The infusions administered to participants were visually and sensorially similar and coded. No deviations from the protocol were noted during the study. Similarity and codification minimized the risk of systematic failure in conducting the study.
Bias due to missing outcome data	LOW	All participants completed the study and there were no dropouts. The results tables present pre- and post-study data for each group of participants. The authors found no evidence of selective omission of data due to dropouts.
Bias in measurement of the outcome	LOW	The parameters are objective (TT, HR, pulse oximetry, hematological blood markers) and were measured using standardized methods. Blinding the individuals responsible for assessing the parameters reduced the risk of bias.
Bias in selection of the reported result	HIGH	This publication does not provide information on trial registration (no number in databases such as ClinicalTrials.gov or UMIN) or the research protocol.

Final score:

× **High risk of bias:** Despite a solid experimental design, the study is compromised by low transparency and selective reporting, as there is no clinical registration or protocol.
